# Risk of postpartum depressive symptoms is influenced by psychological burden related to the COVID-19 pandemic and dependent of individual stress coping

**DOI:** 10.1007/s00404-022-06854-0

**Published:** 2022-12-08

**Authors:** Sarah Meister, Eva-Maria Dreyer, Laura Hahn, Marilena Thomann, Lucia Keilmann, Susanne Beyer, Clarissa Mayer, Gwendolin Prins, Uwe Hasbargen, Sven Mahner, Udo Jeschke, Thomas Kolben, Alexander Burges

**Affiliations:** 1 Department of Obstetrics and Gynecology, University Hospital, LMU Munich, Marchioninistr. 15, 81377 Munich, Germany; 2 Faculty of Psychology, University Würzburg, Würzburg, Germany; 3 Faculty of Humanities and Social Sciences, University of Augsburg, Augsburg, Germany; 4 Department of Gynecology and Obstetrics, University Hospital Augsburg, 86156 Augsburg, Germany

**Keywords:** Postpartum depression, COVID-19 pandemic, Mental disorders, Stress and coping inventory

## Abstract

**Purpose:**

There are different studies worldwide, which have shown a higher risk of mental disorders due to the COVID-19 pandemic. One aim of this study was to identify influencing factors of the psychological burden related to the COVID-19 pandemic and the impact on the development of postpartum depression. Further, the role of individual stress and coping strategies was analyzed in this context.

**Materials and methods:**

Between March and October 2020, 131 women in obstetric care at the LMU Clinic Munich completed a questionnaire at consecutive stages during their perinatal period. The times set for the questionnaire were before birth, 1 month, 2 months, and 6 months after birth. The questionnaire was designed to evaluate the psychological burden related to the COVID-19 pandemic. For this a modified version of the Stress and coping inventory (SCI) and the Edinburgh Postnatal Depression Scale (EPDS) was used.

**Results:**

We could show that the psychological burden related to the COVID-19 pandemic influenced the EPDS score 1, 2 and 6 months after birth. In addition, the prenatal stress and individual coping strategies affected the EPDS and the burden related to the COVID-19 pandemic before and after birth significantly.

**Conclusion:**

An association of the psychological burden related to the COVID-19 pandemic with the risk of developing postpartum depressive symptoms could be shown in this study. In this context, the separation of the partner and the family was recognized as an important factor. Furthermore, the SCI was identified as an effective screening instrument for identifying mothers with an increased risk of postpartum depression. Hereby allowing primary prevention by early intervention or secondary prevention by early diagnosis.

**Supplementary Information:**

The online version contains supplementary material available at 10.1007/s00404-022-06854-0.

## What does this study add to the clinical work

**Table:** 

Our data underline that the psychological burden related to the COVID-19 pandemic is clearly associated with the risk of postpartum depressive symptoms and that stress and coping profiles are associated with the vulnerability for the risk of postpartum depressive symptoms and high burden related to the COVID-19. Screening for coping profiles and mental stress before and after delivery would be necessary to address psychological burden by targeting intervention strategies for the prevention of long-term impacts on maternal well-being and child development.	

## Introduction

Different studies, which have been performed worldwide during the COVID-19 pandemic showed an increased risk for mental disorders [1]. In the general population of different countries, relatively high rates of anxiety, depression, post-traumatic stress disorders, and psychological distress were found during the COVID-19 pandemic [2–4]. Different risk factors for the development of such diagnoses have been identified e.g. female gender, age ≤ 40 years, presence of chronic or psychiatric illnesses, unemployment, student status and frequent exposure to social media [3]. Specifically for the German population, data indicate a negative effect of the COVID-19 situation and the accompanying restrictions on mental health, showing an increase in depressive and anxiety symptoms [5]. For pregnant women, restrictions and consequences on the daily life, as well as the uncertainty about regulations at the hospitals during and after delivery, represented crucial risk factors for COVID-19 related psychological burden [6]. With the outbreak of the COVID-19 pandemic, the inexperience and vagueness regarding possible consequences of a prepartum infection and potentially negative effects on the unborn baby, seem likely to influence the psychological burden. Different studies which have investigated the occurrence of depressive symptoms and anxiety of delivery during the COVID-19 pandemic, revealed an increased incidence of postpartum depression [7, 8].

After delivery many mothers develop a psychological disorder. Around 25% of these women suffer from postpartum dysphoria in the first weeks after delivery, named baby blues [9]. Further 10–15% of the newly mothers develop a postpartum depression (PPD) requiring treatment, [10, 11] this number was even increased by 30–40% during the COVID-19 pandemic [12]. A PPD is characterized by a depressive mental state with listlessness, exhaustion joylessness, loss of interest, concentration disorders, anxiety as well as feelings of guilt and suicidal thoughts [13]. PPD is frequently recognized and treated very late or not at all. However, this mental illness causes severe problems, not only for the mother but also for the child [14] in terms of difficulties in cognitive functioning and social contact with parents, as well as poor self-control [15]. Further, the mothers depression might affect the relationship to her baby [16]. Accordingly, screening of PPD and the development of distinct screening methods is of high interest and very important to avoid potential harm to mother and child. Mothers with a lack of social support suffered from PPD more often than mothers with a well-established social life [17, 18]. Further a disturbed partnership enhances the occurrence of PPD [19]. Various predictors, like a personal history of previous depressive episodes and anxiety before delivery, as well as significant psychosocial stressors during childcare, like fatigue, and lack of sleep, are associated with PPD [20]. When considering stressors and their influence on the development of PPD the importance of individual stress management-named stress coping-must be emphasized. Inadequate coping due to e.g. missing social support leads to negative stressors [21]. Therefore, one important aspect that needs to be analysed, is how individual coping is affecting the prevalence of postpartum depressive symptoms and PPD during the COVID-19 pandemic.

One aim of this study was to investigate the psychological burden related to the COVID-19 pandemic and the main contributing factors by a questionary survey. In addition, we wanted to assess how the strain related to the COVID-19 pandemic influences the occurrence of postpartum depressive symptoms. Further we wanted to address the question of how individual coping strategies and stress are contributing to the risk of developing postpartum depressive symptoms and whether it correlates with the psychological burden related the COVID-19 pandemic. Therefore, we tested the Stress and coping inventory (SCI) as a screening instrument to categorize study participants into different groups of vulnerability, to identify women with a higher risk for postpartum depressive symptoms and higher strain related to the COVID-19 pandemic.

By addressing the individual stress level and coping strategies, possible approaches as how to decrease the risk of developing postpartum depressive symptoms and PPD might be found. Findings about distinct screening instruments might enable to avoid or reduce that risk through intensive individual support during and after birth.

## Methods

### Study design

We performed a prospective cohort study by following up a group of pregnant women during their perinatal period. The follow-up took place prepartum, peripartum and postpartum by a questionary survey (prenatal, 1, 2 and 6 months postpartum). So, a longitudinal analysis with different printed questionnaires was performed. Detailed information about the questionnaires is given in the following paragraphs.

**Fig. 1:**
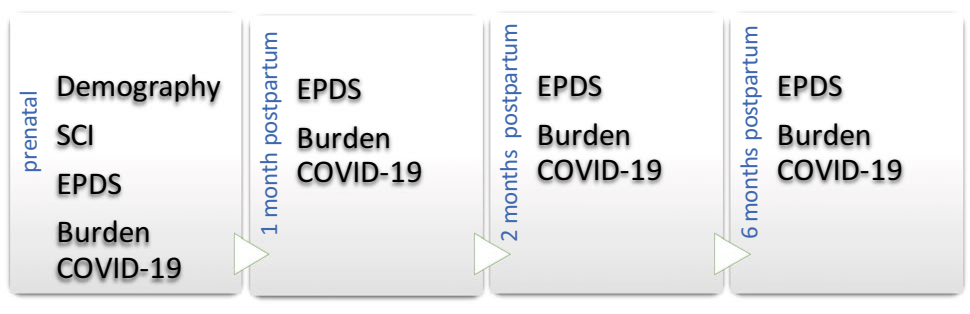
Schematic representation of the survey time points and the used questionaries: Stress-Coping-Inventory (SCI), Edinburgh-Postnatal-Depression-Scale (EPDS)

The survey was performed by self-report questionnaires which were handed out to participants at the time points related to childbirth as presented in Fig. 1. The questionaries were all presented in the same order for all participants, to avoid a possible bias.

### Study population

The study was conducted with 142 pregnant women, who were under obstetric care between the 23rd of March 2020 and the 22nd of October 2020 at the LMU Perinatal Center Grosshadern of the Department of Obstetrics and Gynaecology. Due to exclusion criteria such as not having completed all the questionnaires at all intervals needed for the analysis, 11 women were excluded from the study. Due to the sudden beginning of the COVID-19 pandemic, 35 of the 142 participants of the cohort were retrospectively included into the study. All participants of the monocentric study fulfilled the subsequent criteria: a due date (spontaneous or caesarean section) within the next 48 h, being 18 years or older and written consent.

Demographic data are depicted in Table 1. Further clinical data like mode of delivery and whether there were complications during delivery were evaluated. All participants gave their written consent for participation and completed the questionnaires in printed form. The present study was approved by the local ethics committee of the Ludwig Maximilian University of Munich (reference number–Nr. 20–378).

**Table 1:** Demographic and clinical patients’ characteristics

Variables	*n *(%)*, N* = *131*	
Age	19–34	77 (58.8)
	35–48	51 (38.9)
	Missing	3 (2.3)
Relationship status	Single Married Divorced	26 (19.8) 102 (77.8) 3 (2.4)
School-leaving qualification	None Main school Middle maturity Highschool Diploma	2 (1.5) 11 (8.4) 27 (20.6) 91 (69.4)
Pregnancy	Primiparous	58 (44.3)
	Multiparous	71 (54.2)
	Missing	2 (1.5)
Prior miscarriages	0 1 2 3 Missing	108 (82.4) 10 (7.6) 5 (3.8) 2 (1.5) 6 (4.6)
Complications^1^	Yes	49 (37.4)
	No	82 (62.6)
Mode of delivery	Vaginal delivery	74 (56.5)
	Caesarean section	43 (32.8)
	Missing	14 (10.7)

^1^Suspect/path. CTG green amniotic fluid, umbilical cord entanglement, protracted birth, fever sub partu

### Questionnaires

#### Stress and coping inventory (SCI) (modified)

The stress and coping inventory (SCI) is a German-language stress questionnaire with 54 items [22]. The first 21 items of the SCI are organized in three subscales consisting of seven items each: “stress caused by insecurity”, “stress caused by being overwhelmed” and “stress caused by loss”. Here, a seven-point Likert scale from “not burdened” to “very heavily burdened” is operated. Together, these three subscales assess the total stress level in the last three months. The following 13 items measuring physical stress symptoms, were not used as in pregnancy these values are biased by physical burdens due to pregnancy. The last 20 items were used to evaluate the coping strategies with a four-point Likert scale (“positive coping”, “active coping”, “coping by support”, “coping by believing in God or powers that be” and “coping by drinking alcohol and/or smoking”) being applied on these items. For the evaluation of SCI scales, the sum of all item points of each scale was formed following the instructions of the evaluation manual.

To organize the cohort into different profiles the evaluation manual was used. The median of the actual stress burden (stress due to uncertainty + stress due to overload + stress due to loss + overall stress) and of the adaptive stress coping (positive thinking + active coping with stress + social support + support in God faith) was used (median_stress_ = 29, median_coping_ = 43). The groups are structured as followed: profil A - high stress level with poor coping (median_stress_ > 29, median_coping_ < 43), profil B - high stress level with effective coping (median_stress _> 29, median_coping _> 43), profil C - low stress level with poor coping (median_stress_ < 29, median_coping_ < 43), profil D - low stress level with effective coping (median_stress_ < 29, median_coping_ > 43). In Supp. Table 1, you can find the distribution and division of our study sample in the different profiles.

#### Depressive symptoms (EPDS)

The Edinburgh Postnatal Depression Scale (EPDS) was developed in 1987 as a screening instrument for postnatal depression and translated and adapted to German [23–25]. The total score is the sum of all ten items with a four-point Likert scale (from 0 to 3). An EPDS value of 10 or higher has a middle (10–12) to high (> / = 13) probability for depression [26]. Patients were asked to answer how they felt in the last 7 days.

#### Burden related to the COVID-19 pandemic

To evaluate the psychological burden related to the COVID-19 pandemic, questionnaires were created where the general psychological burden due to the COVID-19 pandemic (“How much did the COVID-19-pandemic strained you psychologically ?”) was analyzed by a four-point Likert scale from “very low”, “low”, to “high” and “very high” before childbirth, in the first month after birth, in the second month after birth, between the 2nd and 6th month and in the 6th month after birth. Further, following influencing factors were evaluated by the same Likert scale. Patients were asked: “Which factors lead to this psychological burden in what extent?” in the shape of four-point Likert scale with following answer options: “possible consequences of infection for your child”, “possible consequences of infection for you”, “possible separation from the child after birth”, “separation from family members during pregnancy”, “separation from the partner before birth”, “separation from partner during birth”, “possible consequences for the time after the birth (lack of direct care by a follow-up midwife, contact restrictions)”, “Separation from family members after birth”, "restrictions of your leisure activities due to the COVID-19 pandemic”, “lack of direct contact and exchange with friends”, “current tendency of the infection course”. In Supp. Table 2, you can find the overview of the distribution of our study sample and at which time point of retrieval which answer possibilities were presented. This questionnaire was designed at the beginning of the COVID-19 pandemic by our group. Therefore, it is no standardized questionnaire. For reliability analysis, Cronbach's alpha was calculated to assess the internal consistency of the questionnaire, which was satisfying (Supp. Table 2).

### Statistical analysis

Software SPSS Statistics 26 (IBM in New York, USA) was used to perform statistical analyses and table creation. Data are presented accordingly as mean (± standard deviation [SD]) or median (interquartile percentile) values. Distribution analysis was performed by the Shapiro–Wilk test. To examine whether the EPDS varies at different times of retrieval in demographic and birth-related categories the non-parametric Mann–Whitney–U (complications/birthmode) and Kruskal–Wallis Test (relationship status/leaving school qualification/pregnancy/abortions) was performed. To compare non-parametric distributed means in measurement repetitions, such as burden related to the COVID-19 pandemic or the EPDS at different time points of retrieval, the non-parametric Friedman's two-factor ANOVA was used. We performed a Spearman-Rho-correlation to examine a connection between the EPDS and the psychological COVID-19-burden. Multiple regression was performed to predict the influence of the SCI and the psychological burden due to COVID-19 on the EPDS. Study sample distribution in the SCI-groups was tested by the Kruskal–Wallis Test. The non-parametric Chi-squared test was used to show statistical dependence between categorial variables (e.g. SCI-Groups and EPDS in categories low/medium/high risk for depression). Distribution analysis was Bonferroni-corrected. *P* values ≤ 0.05 were rated as statistically significant.

We performed a sample size calculation before starting the survey. Assumption of minimum incidence of postpartum depressive symptoms was set to 3%, maximum incidence to 20%. Alpha-cut off was set to 0.05 with a power of 0.8. The statistical output indicated that a design with 54 samples per group (a total of 108) has a ~ 80% chance to detect a difference of 0.1

## Results

### Impact of demographic factors and clinical characteristics on the EPDS

58.8% of the participants were between 18 and 34 years old, 38.9% between 35 and 48 years. 77.8% were married (Table 1). Further school-leaving qualification was assessed. There, the majority of the participants had a high school diploma (69.4%). 54.2% were multiparas and the majority did not have prior miscarriages (82.4%). In 37.4% of the pregnancies, complications were documented, most of the deliveries were vaginal (56.5%). Spearman Rho correlation was performed to analyse the impact of demographic factors and clinical characteristics (relationship status, school-leaving qualification, pregnancy, prior miscarriages, complications) on the EPDS. The data show no significant difference of the EPDS in relation to demographic factors (age, relationship status, school-leaving qualification) prenatal and 1 month after birth (Table 2). The maternal age did not correlate significantly with the EPDS (prenatal *r* = 0.122, *p* = 0.249; 1 month postpartum: *r* = 0.070, *p* = 0.528; 2 months postpartum *r* = 0.027, *p* = 0.776; 6 months postpartum *r* = 0.136, *p* = 0.182). The EPDS two and 6 months after birth vary significantly in patients who experienced complications during birth, like suspect/pathologic CTG, green amniotic fluid, umbilical cord entanglement, protracted birth, fever sub partu, (2 months postnatal: *r* = − 0.227, *p* = 0.014, 6 months postnatal: *r* = − 0.231, *p* = 0.021).

**Table 2:** Correlations between patients’ demographic and clinical data and the EPDS at different time points of retrieval: the upper value is the correlation coefficient *r*, the second value is the *p* value

EPDS	Prepartum	1 month postpartum	2 months postpartum	6 months postpartum
Correlation coefficient *r p* value	*N* = *94*	*N* = *84*	*N* = *117*	*N* = *99*
Age	− 0.122 0.247	− 0.070 0.528	0.027 0.776	0.133 0.191
Relationship status	− 0.017 0.872	− 0.108 0.328	− 0.018 0.849	− 0.073 0.470
School-leaving qualification	0.063 0.542	0.109 0.325	0.049 0.596	0.136 0.176
Pregnancy	− 0.197 0.057	− 0.275* 0.012	− 0.069 0.431	− 0.051 0.615
Prior abortions	0.217* 0.036	0.060 0.592	0.075 0.431	0.074 0.476
Complications^1^	− 0.170	− 0.094	− 0.227*	− 0.231*
	0.100	0.394	0.014	0.021

*Significant (*p* < 0.05), **highly significant (*p* < 0.01)

### EPDS and correlation with the burden related to the COVID-19 pandemic

**Fig. 2:**
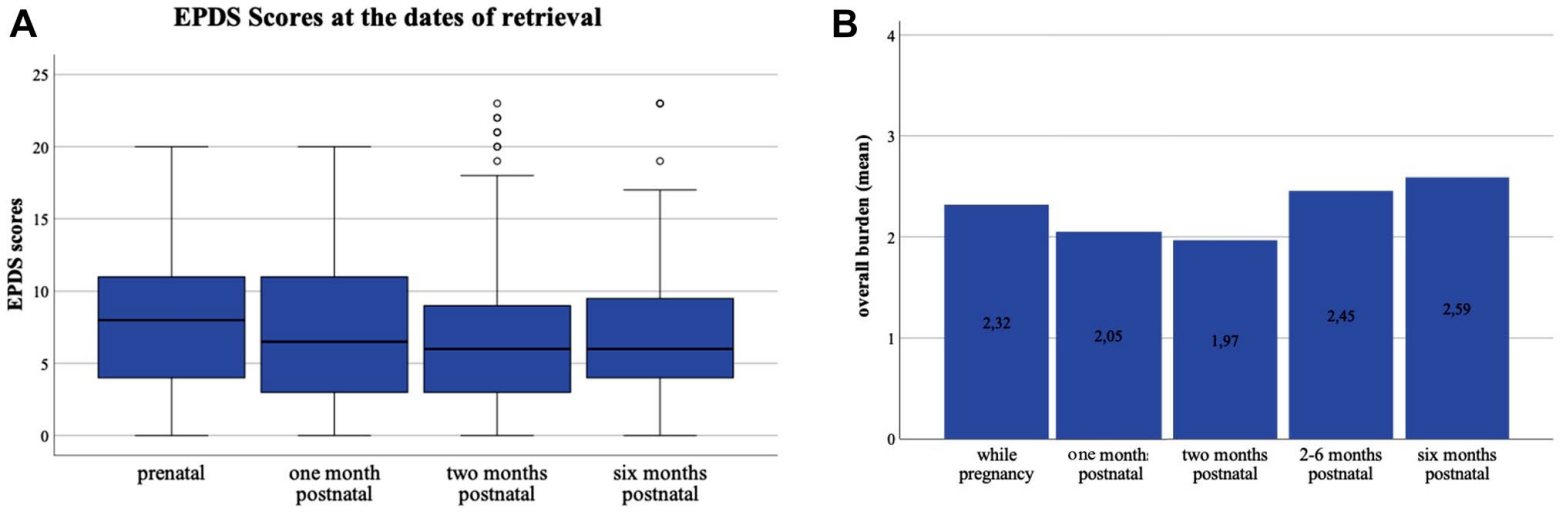
EPDS scores and psychological burden related to the COVID-19 pandemic at time points of inquiry, **A** Boxplots of medians ± SD at the different time points of evaluation, (*N* = 131), **B** means of the psychological burden related to the COVID-19 pandemic

The EPDS was evaluated in the study population prenatally, 1 and 2 months postpartum, as well as 6 months after delivery. A significant difference was found between the different evaluated time points (*p* = 0.021; prenatal = 7.52 ± 4.683 median = 8; 1 month postpartum = 7.20 ± 4.921 median 6.5; 2 months postpartum = 6.84 ± 5.654 median = 6; 6 months postpartum = 6.94 ± 5.101 median = 6) (Fig. 2A). Adjusted according to Bonferroni, there is no clear trend, except from the significant difference between the relatively high EPDS prenatal and the low EPDS 2 months postpartum (*p* = 0.045).

Further, the overall burden related to the COVID-19 pandemic at the different time points related to childbirth was analysed. Significant differences of the mean burden related to the COVID-19 pandemic were found even though there was no obvious trend. An exception being a slight increase of the burden between 2 and 6 months postpartum, as well as an increase of the median over the entire time (*p* < 0.001; prenatal = 2.32 ± 0.938 median = 2; 1 month postpartum = 2.01 ± 2.00 median 2; 2 months postpartum = 1.97 ± 0.870 median = 2; 2–6 months postpartum = 2.45 ± 0.918 median = 2; 6 months postpartum = 2.589 ± 0.8437 median = 3) (Fig. 2B, Supp. Table 3).

**Fig. 3:**
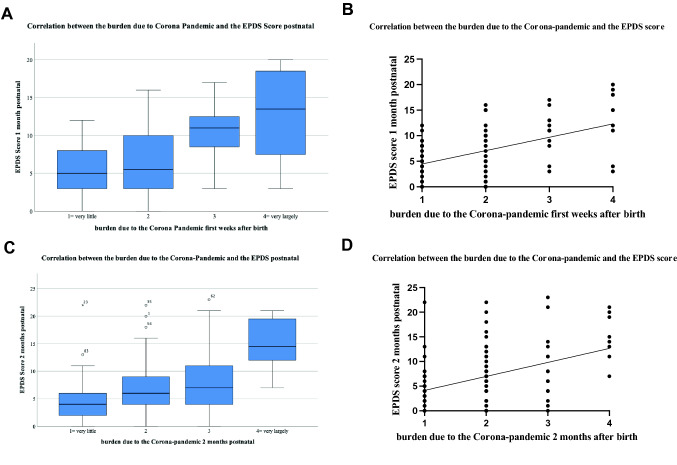
Correlation between the burden related to the COVID-19 pandemic and the EPDS: **A** Boxplots EPDS 1 month postpartum and the burden in the first weeks after birth, mean ± SD; **B** Visualization of the correlation EPDS 1 month postpartum and the burden in the first weeks after birth, correlation was calculated using the Spearman-Rho-Correlation-Test; **C** Boxplot of the correlation between EPDS 2 months postpartum and the burden in the 2 months after birth, mean ± SD; **D** Visualization of the correlation EPDS 2 months postpartum and the burden in the two months after birth. Correlation was calculated using the Spearman-Rho-Correlation-Test. The regression line refers to the total collective

To reveal possible associations between the psychological burden related to the COVID-19 pandemic, a correlation analysis with the EPDS was performed. We found a strong positive correlation of the EPDS score and the overall psychological burden related to the COVID-19 pandemic prenatal, 2–6 months postpartum (*r* = 0.411, *p* = 0.00) and 6 months postpartum (*r* = 0.400, *p* = 0.01). The EPDS 1 month postpartum strongly correlated with the perceived burden at 1 month (*r* = 0.416, *p* < 0.001), 2 months (*r* = 0.459, *p* < 0.001), 2–6 months (*r* = 0.407, *p* = 0.001) and 6 months postpartum (*r* = 0.461, *p* < 0.001). 2 months postpartum a strong positive correlation of the EPDS and the burden related to the COVID-19 pandemic could be found 2–6 (*r* = 0.411, *p* < 0.001) and 6 months (*r* = 0.419, *p* < 0.001) postpartum. 6 months after delivery the EPDS correlated merely positively with the burden due to the COVID-19 pandemic 2 (*r* = 0.397, *p* < 0.001) and 6 months (*r* = 0.330, *p* < 0.001) postpartum (Fig. 3, Table 3).

**Table 3:** Correlations between the psychological burden related to the COVID-19 pandemic and the EPDS scoring: the upper value is the correlation coefficient *r*, the second value is the *p *value

	Prenatal	1 month postpartum	2 months postpartum	2 to 6 months postpartum	6 months postpartum
EPDS score prenatal	0.335** 0.002	0.327** 0.003	0.306** 0.005	0.411** < 0.001	0.400** 0.001
EPDS score 1 month postpartum	0.265* 0.021	0.416** < 0.001	0.459** < 0.001	0.407** 0.001	0.461** < 0.001
EPDS score 2 months postpartum	0.251** 0.007	0.369** < 0.001	0.373** < 0.001	0.411** < 0.001	0.419** < 0.001
EPDS score 6 months postpartum	0.168 0.106	0.182 0.080	0.277** 0.007	0.397** < 0.001	0.330** 0.001

Overall psych. Burden related to the COVID-19 pandemic

*Significant (*p* < 0.05), **highly significant (*p* < 0.01)

Furthermore, we performed a multiple regression analysis, showing the influence of the burden related to the COVID-19 pandemic on the EPDS Score at all survey time points (prenatal (*R*^2^ = 0.169, *p* = 0.013), 1 month (*R*^2^ = 0.239, *p* = 0.002), 2 months (*R*^2^ = 0.180, *p* = 0.001) and 6 months (*R*^2^ = 0.121, *p* = 0.025) postpartum).

Further, the influence of different factors on the burden related to the COVID-19 pandemic were analyzed by correlation and multiple regression. Correlations are shown in Table 4. By performing multiple regression, we found a significant influence of the separation from the partner before delivery on the EPDS prepartum (*p* = 0.022). In addition, the prenatal fear of being separated from relatives influenced the EPDS 2 months postpartum (*R*^2^ = 0.279, *R*^2^ = 0.253, *p* = 0.014) significantly.

**Table 4:** Correlations between specific psychological burdens related to the COVID-19 pandemic and the EPDS scoring at different times of retrieval: the upper value is the correlation coefficient *r*, the second value is the *p *value

EPDS	Prepartum	1 month postpartum		2 months postpartum	6 months postpartum	
Which factors have led to mental burden related to the COVID-19 pandemic? *r p* value	Prepartum	First days after birth	1 month postpartum	2 months postpartum	2–6 months postpartum	6 months postpartum
Possible consequences of infection for your child	0.252* 0.023	0.533** < 0.001	0.528** < 0.001	0.318** < 0.001	0.096 0.348	0.168 0.099
Possible consequences of infection for you	0.252* 0.023	0.475** < 0.001	0.470** < 0.001	0.318** < 0.001	0.155 0.128	0.220* 0.030
Possible separation from the child after birth	0.196 0.080					
Possible consequences for the birth	0.222* 0.047					
Separation from family members during pregnancy	0.265* 0.017					
separation from the partner before birth	0.401** < 0.001					
Separation from the partner during birth	0.313** 0.004					
Separation from the partner after birth		0.338** 0.003				
Possible consequences for the time after the birth (lack of direct care by a follow-up midwife, contact restrictions)	0.318** 0.004		0.425** < 0.001	0.299** 0.002	0.228* 0.024	0.300** 0.003
Separation from family members after birth	0.280* 0.011	0.437** < 0.001	0.506** < 0.001	0.442** < 0.001	0.230* 0.023	0.282** 0.005
Restrictions of your leisure activities due to the COVID-19 pandemic	0.267* 0.016	0.171 0.136	0.284* 0.012	0.260** 0.006	0.078 0.445	0.133 0.192
Lack of direct contact and exchange with friends	0.272* 0.014	0.187 0.103	0.246* 0.031	0.274** 0.004	0.137 0.180	0.214* 0.034
Current tendency of the infection course						0.217* 0.032

* Significant (*p* < 0.05), ** highly significant (*p* < 0.01)

### The SCI as possible screening instrument for postpartum depressive symptoms

**Fig. 4:**
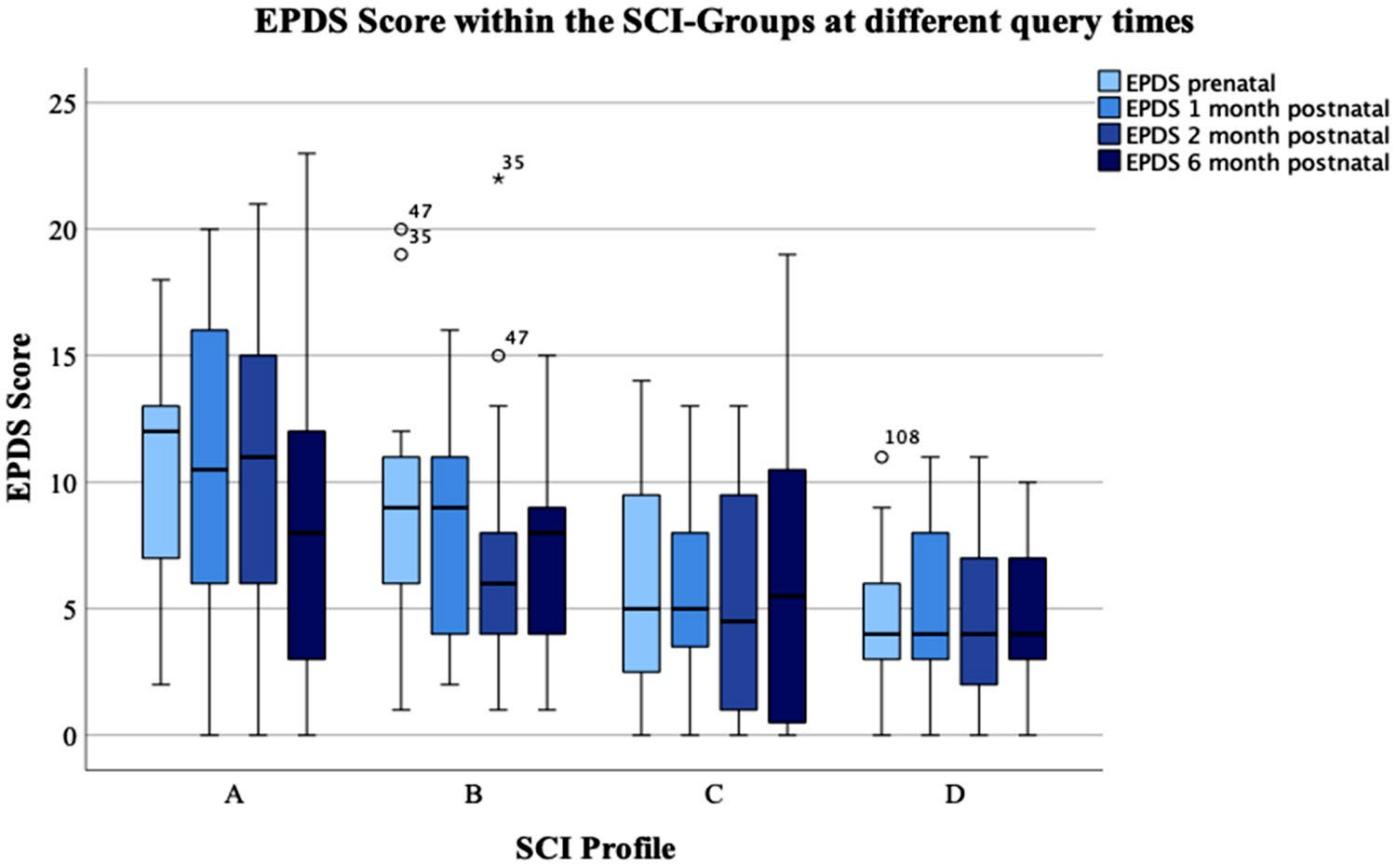
EPDS evaluated in the different SCI profiles, Boxplot of the EPDS scores mean ± SD in the different SCI profiles (**A**, **B**, **C**, **D**)

To evaluate whether the SCI could be used as instrument to assess the risk for postpartum depressive symptoms prenatally, we analysed the EPDS in the different SCI profiles. The Boxplot in Fig. 4 shows the EPDS-Scores in the 4 different SCI-Groups at the different survey times points.

First of all, the mean EPDS scores in the different SCI profiles were compared with each other. This revealed significant differences between the EPDS scores and the four different SCI profiles at all time points (prepartum: *p* = 0.001, 1 month postpartum: *p* = 0.014, 2 months postpartum: *p* = 0.001, 6 months postpartum: *p* = 0.025). Further analysis with pairwise comparisons showed that these differences especially occurred between the SCI profile A and D (prepartum: *p* = 0.001, 1 month postpartum: *p* = 0.050), A and C (prepartum: *p* = 0.002, 1 month postpartum: *p* = 0.025, 2 months postpartum: *p* = 0.003) and B and C (after 2 months postpartum: *p* = 0.029).

Performing the Chi-square test at the different survey time points, showed a statistically significant dependence of the EPDS categories (chance for postpartum depression low / medium/high) on the SCI profiles prepartum (Chi^2^ = 21.595, *p* = 0.001), 1 month (Chi^2^ = 16.337, *p* < 0,05) and 2 months (Chi^2^ = 15.451, *p* < 0.05) postpartum.

By performing linear regression analysis, a significant influence of the SCI-Score on the EPDS prepartum (*R*^2^ = 0.154, *p* < 0.001), 1 month (*R*^2^ = 0.131, *p* = 0.001), 2 (*R*^2^ = 0.137, *p* < 0.001) and 6 (*R*^2^ = 0.062, *p* = 0.017) months postpartum could be revealed. Analysing the influence of the mean total stress—assessed by the SCI—on the EPDS showed a significant influence on the EPDS prenatal (*p* < 0.001), 1 month (*p* < 0.001), 2 months (*p* < 0.001) and 6 months (*p* = 0.006) postpartum.

### Impact of the SCI score on the burden related to the COVID-19 pandemic

**Fig. 5:**
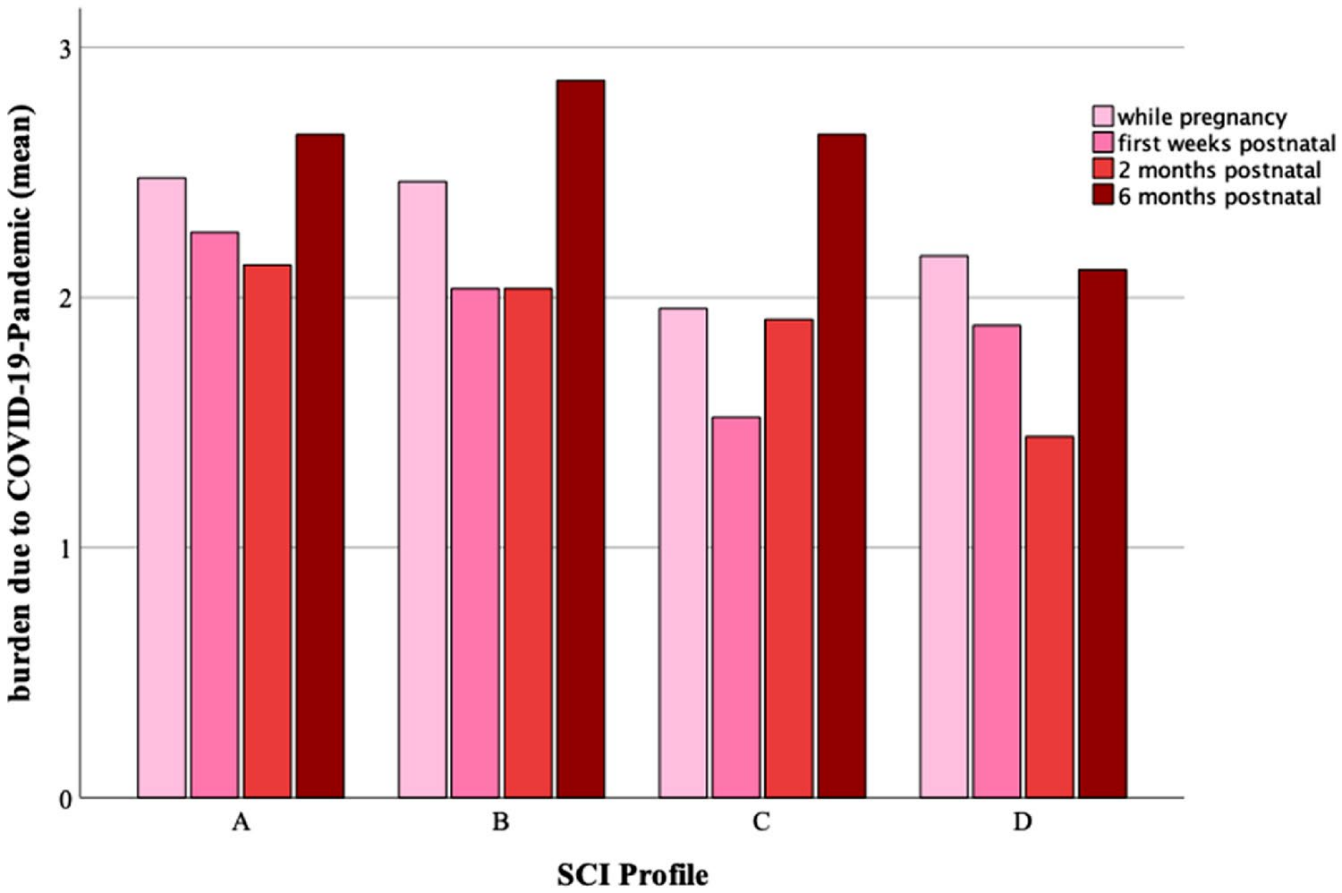
The mean burden related to the COVID-19 pandemic in the different SCI profiles

We further examined the burden related to the COVID-19 pandemic in the different SCI groups (Fig. 5). Further, significant differences of the overall psychologic burden related to the COVID-19 pandemic between the different SCI profiles could be found during pregnancy (*p* = 0.037), 1 month (*p* = 0.009), 2 (*p* = 0.030) and 6 (*p* = 0.030) months postpartum (Fig. 5, Supp. Table 4 and 5).

Interestingly we found a significant influence of the SCI-Scoring on the overall burden related to the COVID-19 pandemic during pregnancy (*R*^2^ = 0.126, *p* < 0.001), 1 month (*R*^2^ = 0.076, *p* < 0.05), 2 (*R*^2^ = 0.134, *p* < 0.001) and 6 (*R*^2^ = 0.065, *p* < 0.05) months postpartum, by performing multiple regression analysis.

Concerning the evaluated factors leading to the mean overall burden related to the COVID-19 pandemic, we could find significant differences in the SCI-profiles, especially between profile A and D or A and C, meaning increased scores in profile A compared to C and D. There, especially the separation from the partner or family members during pregnancy or after birth played an important role. Further restrictions of leisure activities and the missing contact with friends stressed patients in profile A significantly more than in profile C or D (Supp. Table 3).

## Discussion

At the beginning of the COVID-19 pandemic, the missing knowledge about the virus and its dangers in pregnancy, resulted in a great uncertainty and anxiety, especially for pregnant women [27]. In this study, we could demonstrate that the psychological burden related to the COVID-19 pandemic was clearly associated with the risk of postpartum depressive symptoms. However, the mode of delivery and demographic factors did not influence the risk of postpartum depressive symptoms significantly, the occurrence of complications during birth, like stress to the unborn did. Further, we identified the SCI as an effective instrument, to screen mothers before delivery and subdivide them into groups—with an unfavourable constellation of stress and coping strategies, having a higher risk to suffer from postpartum depressive symptoms compared to those who had favourable constellation of stress and coping strategies, with a lower risk for PPD symptoms.

PPD is a severe and very frequently appearing disorder which occurs within weeks and months after delivery. During the COVID-19 pandemic several studies indicated an increasing incidence of PPD, which was highly related to stress and anxiety during pregnancy, delivery and postpartum [12, 28, 29]. In our study sample which was followed over 6 months, the mean EPDS differed significantly over the time points of retrieval. Exceptionally high EPDS values prepartum might indicate the particularity of the situation due to the COVID-19 pandemic. In contrast to other data which indicate a higher incidence of PPD in mothers who underwent caesarean sections we did not find a significant correlation with the delivery mode and the EPDS in our study [30–32]. Further, demographic factors could not be revealed as relevant risk factors in our study, conversely to the path analysis model, which showed a significant influence of the maternal age, occupation, living conditions and quality of life on PPD [33]. However, complications as pathologic CTG or green amniotic fluid—meaning increased stress to the unborn during birth—seemed to play a significant role, which might have led to a more stressful birth experience.

The trends of the changing psychological burden related to the COVID-19 pandemic were similar to the EPDS assessed over the time. Participants with a high psychological burden related to the COVID-19 had high EPDS values. An association could be confirmed by the means of correlation analysis between the overall mean psychological burden related to the COVID-19 pandemic and the EPDS, where the psychological burden particularly in month 1 and 2 months postpartum seemed to have a relevant influence on the EPDS 1, 2 and 6 months postpartum. Multiple regression analysis showed that the burden related to the COVID-19 pandemic influenced the EPDS significantly. The main factors influencing the psychological burden related to the COVID-19 pandemic changed over time, as prenatally and in the first days after delivery the separation from the partner and the family, as well as possible consequences for the delivery were superficial and correlated highly significant with the EPDS. These data are going along with a recent publication, showing increased anxiety and depression rates as well as postpartum traumatic stress symptoms, when mothers were unaccompanied during birth [34]. In contrast to our findings their observations showed higher levels of anxiety and trauma scores and lower well-being in women with caesarean sections, which was aggravated by visitation restrictions after birth.

In our study the separation from family and friends was one of the main factors for concern 1 month and 2 months postpartum. Later, the risk of an infection of the infant with SARS-CoV-2 was one important concern (Supp. Table 2).

Anxiety, stress and ineffective coping are essential factors influencing the risk of postpartum depressive symptoms. There are several studies which have demonstrated the influence of coping strategies on the development of PPD, independently from the COVID-19 pandemic. There, active coping (emotional support, positive reframing and acceptance) was shown to predict depressive symptoms [35]. Our findings confirmed that high stress levels and unfavourable coping strategies increased the risk of postpartum depressive symptoms. Interestingly the stress level seemed to play a very important role in our cohort. The occurrence and the effects of PPD are underestimated because of the high number of unrecorded cases of suffers. PPD often remains undiscovered and untreated [36]. Therefore, the introduction of an effective screening instrument to identify patients with a high risk for the development of PPD symptoms is highly relevant to prevent postpartum depressive symptoms and PPD. This becomes even more important  when considering the expected chronic mental burden related to the COVID-19 pandemic. Since peripartum stress represents a very important factor and coping is crucial for the development of PPD [37], the SCI was tested as possible screening element for PPD predisposition in our study. We classified our cohort into 4 different profile groups with high stress level and poor coping (profile A), high stress level and effective coping (profile B), low stress level and poor coping (profile C) and low stress level with effective coping (profile D). Our data revealed a clear correlation of the different profiles with the occurrences of PPD symptoms. Further, the burden related to the COVID-19 pandemic and the EPDS were dependent on the SCI score. This points to a promising opportunity to use the SCI as screening instrument to identify mothers with high vulnerability to stress, psychological burden and postpartum depressive symptoms.

At this point, some limitations of this study need be considered when interpreting our results: the small size of the study group and the missing references of the designed COVID-19 questionnaire to evaluate the psychological burden related to the COVID-19 pandemic need to be considered. Further, the study is based on a survey with printed questionnaires, rather than personal interviews.

Our data underline that the psychological burden related to the COVID-19 pandemic is clearly associated with the risk of postpartum depressive symptoms. Evaluation of stress and coping profiles revealed the vulnerability for the risk of postpartum depressive symptoms and high burden related to the COVID-19 pandemic in our study sample. Therefore, individual support to improve coping strategies might have a positive effect on the mean mental strain and the COVID-19 pandemic related burden. Mental health screening before and after delivery would be necessary to address psychological burden by targeting intervention strategies for the prevention of long-term impacts on maternal well-being and child development.

## Supplementary Information

Below is the link to the electronic supplementary material.

Supplementary file1 (DOCX 16 KB)

Supplementary file2 (DOCX 13 KB)

Supplementary file3 (DOCX 16 KB)

Supplementary file4 (DOCX 14 KB)

Supplementary file5 (DOCX 16 KB)

Supplementary file6 (DOCX 16 KB)
