# Supplementary material for: Risk of postpartum depressive symptoms is influenced by psychological burden related to the COVID-19 pandemic and dependent of individual stress coping
Source: Arch Gynecol Obstet. 2022 Dec 8;308(6):1737–48. doi: 10.1007/s00404-022-06854-0 (PMC9735014; doi:10.1007/s00404-022-06854-0)
Supplement: Supplementary file 2 — Supplementary file2 (DOCX 13 KB) [file 404_2022_6854_MOESM2_ESM.docx]

| ***SCI*** | ***Profile A*** | ***Profile B*** | ***Profile C*** | ***Profile D*** | ***sum*** |
| --- | --- | --- | --- | --- | --- |
| *acute stress* | >58 | | <58 | |  |
| *adaptive coping* | >43 | <43 | >43 | <43 |  |
| *n* | 32 | 31 | 41 | 27 | 131 |
| *%* | 24,43 | 23,66 | 31,30 | 20,61 | 100 |

**Supp. Table 1.** Distribution and classification of the different SCI profiles
