# Supplementary material for: Risk of postpartum depressive symptoms is influenced by psychological burden related to the COVID-19 pandemic and dependent of individual stress coping
Source: Arch Gynecol Obstet. 2022 Dec 8;308(6):1737–48. doi: 10.1007/s00404-022-06854-0 (PMC9735014; doi:10.1007/s00404-022-06854-0)
Supplement: Supplementary file 3 — Supplementary file3 (DOCX 16 KB) [file 404_2022_6854_MOESM3_ESM.docx]

|  | **prenatal** | **first month postpartum** | **2 months postpartum** | **2-6 months postpartum** | **6 months postpartum** |
| --- | --- | --- | --- | --- | --- |
| **burden overall** |  |  |  |  |  |
| *mean (SD)* | 2.32 (0.94) | 2,01 (0,94) | 1,97 (0,87) | 2,45 (0,92) | 2,59 (0,84) |
| **specific factors leading to the burden** |  | | |  |  |
| *mean (SD)* |  | | |  |  |
| possible consequences of infection for your child | 2.40 (1.00) | 1.93 (1.05) | 1.97 (0.99) | 2.26 (1.00) | 2.34 (0.98) |
| possible consequences of infection for you | 1.96 (0.98) | 1.73 (0.91) | 1.74 (0.85) | 2.09 (0.96) | 2.13 (0.97) |
| possible separation from the child after birth | 2.40 (1.14) | - | - | - | - |
| possible consequences for the birth | 2.52 (1.08) | - | - | - | - |
| separation from family members during pregnancy | 2.18 (1.12) | - | - | - | - |
| separation from the partner before birth | 2,46 (1.12) | - | - | - | - |
| separation from partner during birth | 3,02 (1.06) | - | - | - | - |
| possible consequences for the time after the birth (lack of direct care by a follow-up midwife, contact restrictions) | 2.07 (1.04) | 1.68 (1.01) | 1.47 (0.85) | 1.62 (0.98) | 1.59 (0.87) |
| Separation from family members after birth | 2.53 (1.12) | 2.21 (1.13) | 1.83 (0.99) | 2.50 (1.11) | 2.65 (1.06) |
| restrictions of your leisure activities due to the corona pandemic | 2.47 (1.21) | 1.77 (0.99) | 1.83 (0.98) | 2.69 (1.09) | 2.64 (1.01) |
| lack of direct contact and exchange with friends | 2.46 (1.06) | 2.05 (1.04) | 1.81 (0.99) | 2.85 (1.02) | 2.79 (1.00) |
| current tendency of the infection course | - | - | - | - | 2.38 (0.98) |
|  | | | | | |
| ***Cronbachs alpha*** | ***0.842*** | ***0.827*** | ***0.837*** | ***0.620*** | ***0.751*** |

**Supp. Table 2.** Distribution of the different factors leading to the overall psychologic burden related to the COVID-19 pandemic
