# Supplementary material for: Risk of postpartum depressive symptoms is influenced by psychological burden related to the COVID-19 pandemic and dependent of individual stress coping
Source: Arch Gynecol Obstet. 2022 Dec 8;308(6):1737–48. doi: 10.1007/s00404-022-06854-0 (PMC9735014; doi:10.1007/s00404-022-06854-0)
Supplement: Supplementary file 4 — Supplementary file4 (DOCX 14 KB) [file 404_2022_6854_MOESM4_ESM.docx]

| ***Allover burden related to the COVID-19-pandemic*** | *prepartum* | *Days postpartum* | *1 month postpartum* | *2 months postpartum* | *2-6 months postpartum* | *6 months postpartum* |
| --- | --- | --- | --- | --- | --- | --- |
| *Prepartum* | x | 0.117 | 0.057 | 0.013* | 1.000 | 0.699 |
| *Days postpartum* |  | x | 1.000 | 1.000 | 0.004* | <0.001** |
| *Weeks postpartum* |  |  | x | 1.000 | 0.002* | <0.001** |
| *2 months postpartum* |  |  |  | x | <0.001** | <0.001** |
| *2-6 months postpartum* |  |  |  |  | x | 1.000 |
| *6 months postpartum* |  |  |  |  |  | x |

**Supp. Table 3**. Friedman's two-factor ANOVA Analysis. p-values and significances. * significant (p < 0.05), ** highly significant (p < 0.01)
