# Supplementary material for: Risk of postpartum depressive symptoms is influenced by psychological burden related to the COVID-19 pandemic and dependent of individual stress coping
Source: Arch Gynecol Obstet. 2022 Dec 8;308(6):1737–48. doi: 10.1007/s00404-022-06854-0 (PMC9735014; doi:10.1007/s00404-022-06854-0)
Supplement: Supplementary file 5 — Supplementary file5 (DOCX 16 KB) [file 404_2022_6854_MOESM5_ESM.docx]

|  | **prenatal** | **first month postpartum** | **2 months postpartum** | **2-6 months postpartum** | **6 months postpartum** |
| --- | --- | --- | --- | --- | --- |
| **burden overall**  X2 (p) |  |  |  |  |  |
|  | ***8.469 (0.037)**** | ***11.503 (0.009)***** | ***8.953***  ***(0.030)**** | ***9.413***  ***(0.024)**** | ***8.967***  ***(0.030)**** |
| **specific causes**  X2 (p) | | | |  |  |
| possible consequences of infection for your child | *2.2125 (0.547)* | *3.166*  *(0.367)* | *2.582*  *(0.461)* | *2.223*  *(0.528)* | *3.367*  *(0.338)* |
| possible consequences of infection for you | *3.929*  *(0.269)* | *1.425*  *(0.700)* | *0.502*  *(0.918)* | *4.235*  *(0.237)* | ***8.181***  ***(0.042)**** |
| possible separation from the child after birth | *1.302*  *(0.729)* |  |  |  |  |
| possible consequences for the birth | ***10.171 (0.017)**** |  |  |  |  |
| separation from family members during pregnancy | ***9.010 (0.029)**** |  |  |  |  |
| separation from the partner before birth | ***9.612 (0.022)****  ***(0.041)*** |  |  |  |  |
| separation from partner while birth | ***10.601 (0.014)****  ***(0.118)*** |  |  |  |  |
| possible consequences for the time after the birth (lack of direct care by a follow-up midwife, contact restrictions) | *4.743*  *(0.192)* | *3.266*  *(0.352)* | *3.957*  *(0.266)* | *4.459*  *(0.237)* | *4.158*  *(0.245)* |
| Separation from family members after birth | ***7.999 (0.046)**** | ***13.840 (0.003)***** | ***8.134 (0.043)**** | *4.484*  *(0.214)* | *4.254*  *(0.235)* |
| restrictions of your leisure activities due to the corona pandemic | ***15.581 (0.001)***** | ***11.897 (0.008)***** | ***10.618 (0.014)**** | *1.018*  *(0.797)* | *2.876*  *(0.411)* |
| lack of direct contact and exchange with friends | ***21.637 (<0.001)***** | ***18.103 (<0.001)***** | ***13.524 (0.004)***** | *4.384*  *(0.223)* | *6.199*  *(0.102)* |
| current tendency of the infection course |  |  |  |  | *4.912*  *(0.178)* |

**Supp. Table 4.** Factors influencing the psychological burden related to the COVID-19 pandemic in the different SCI Profiles. Upper value is the Kruskal-Wallis X^2^ and lower value represents the p-value * significant (p < 0.05), ** highly significant (p < 0.01).
