# Supplementary material for: Risk of postpartum depressive symptoms is influenced by psychological burden related to the COVID-19 pandemic and dependent of individual stress coping
Source: Arch Gynecol Obstet. 2022 Dec 8;308(6):1737–48. doi: 10.1007/s00404-022-06854-0 (PMC9735014; doi:10.1007/s00404-022-06854-0)
Supplement: Supplementary file 6 — Supplementary file6 (DOCX 16 KB) [file 404_2022_6854_MOESM6_ESM.docx]

| ***How much did the COVID-19-pandemic burden you psychologically?*** | | ***Profile A*** | ***Profile B*** | ***Profile C*** | ***Profile D*** |
| --- | --- | --- | --- | --- | --- |
| ***Prepartum*** | *Very low (1)* | 3 | 6 | 9 | 5 |
|  | *Low (2)* | 11 | 9 | 19 | 9 |
|  | *Strong (3)* | 8 | 8 | 8 | 6 |
|  | *Very strong (4)* | 6 | 6 | 2 | 1 |
|  | *N* | 28 | 29 | 38 | 21 |
| ***Weeks postpartum*** | *Very low (1)* | 4 | 8 | 20 | 7 |
|  | *Low (2)* | 15 | 12 | 12 | 9 |
|  | *Strong (3)* | 3 | 7 | 5 | 3 |
|  | *Very strong (4)* | 6 | 2 | 1 | 2 |
|  | *N* | 28 | 29 | 38 | 21 |
| ***2 Months postpartum*** | *Very low (1)* | 5 | 8 | 14 | 11 |
|  | *Low (2)* | 15 | 12 | 17 | 9 |
|  | *Strong (3)* | 5 | 8 | 5 | 0 |
|  | *Very strong (4)* | 4 | 1 | 2 | 1 |
|  | *N* | 29 | 29 | 38 | 21 |
| ***2-6 months postpartum*** | *Very low (1)* | 2 | 3 | 5 | 4 |
|  | *Low (2)* | 10 | 8 | 9 | 14 |
|  | *Strong (3)* | 10 | 11 | 5 | 3 |
|  | *Very strong (4)* | 3 | 6 | 5 | 0 |
|  | *N* | 25 | 28 | 24 | 21 |
| ***6 months postpartum*** | *Very low (1)* | 1 | 2 | 3 | 3 |
|  | *Low (2)* | 10 | 7 | 7 | 13 |
|  | *Strong (3)* | 11 | 12 | 10 | 5 |
|  | *Very strong (4)* | 3 | 7 | 4 | 0 |
|  | *N* | 25 | 28 | 24 | 21 |

**Supp. Table 5.** Distribution of the study sample in the SCI profiles at the different time points of retrieval
